# Heat and mass transfer of micropolar fluid flow over a stretching sheet by legendre collocation method

**DOI:** 10.1038/s41598-025-10028-8

**Published:** 2025-07-17

**Authors:** K. M. Abdelgaber, Mohamed Fathy, Passant k. Abbassi, R. A. Elbarkoki

**Affiliations:** 1 Department of Physics & Engineering Mathematics, Faculty of Engineering - Mataria, Helwan University, Cairo, Egypt; 2 Faculty of Informatics & Computer Science, The British University in Egypt, Cairo, 11511 Egypt; 3 Basic and Applied Science Department, College of Engineering and Technology, Arab Academy for Science, Technology and Maritime Transport, Cairo, Egypt; 4 Mathematics Department, Faculty of Engineering, German University in Cairo, Cairo, Egypt; 5 Mathematics Department, Faculty of Engineering, Ain Shams University, Abbassia, Cairo Egypt; 6 Mathematics Department, Faculty of Engineering, The Egyptian Chinese University, Cairo, Egypt

**Keywords:** Boundary layer flow, Heat transfer, Thermal radiation, Stretching sheet, Micropolar fluid, Chemical reaction, Magnetic field, Applied mathematics, Fluid dynamics

## Abstract

The integration of micropolar fluid in extrusion processes is critical for understanding and improving the manufacturing of materials that reveal microstructural effects. Extrusion is a commonly used process in sectors such as polymer, food, and metal processing, where a material is pushed through a die to form a product with a desired cross-section (e.g., films, sheets, fibers, tubes). The consequences of magnetic field, thermal radiation, and chemical reaction on the quality of the extruded product constitute a complicated and vital area of research. Hence, the current study is conducted to examine the flow associated with the transport of heat and mass of micropolar fluid across an expandable sheet in the company of an external magnetic field, thermal radiation, and chemical reaction. The problem is controlled by the energy equation for heat transfer, the species transport equation for mass transfer, and the Navier-Stokes equations for momentum. Following some conversions, the subsequent scheme of ordinary differential equations (ODEs) is numerically worked out by applying the Legendre-collocation approach. The velocity, temperature, and concentration profiles are analyzed in relation to the effects of thermal radiation, magnetic field strength, and chemical reaction rate. The findings reveal that the magnetic field will decrease the velocity but on the other hand it will increase the microrotation velocity. The magnetic field and the thermal radiation will enhance the temperature. Finally, the magnetic field will improve the concentration slightly but on the other hand the chemical reaction will decrease it.

## Introduction

The study of flow and the transport of heat and mass above an expandable sheet has significant applications in engineering, especially in the fields of polymer processing, cooling of materials, and bio-fluid dynamics. Micropolar fluids, which have tiny structures like suspended particles or fibers, behave differently from Newtonian fluids because their particles can rotate as they move. Micropolar fluid is another simulation model for the non-Newtonian fluid that exists in a lot of manufacturing processes.

In conjunction with external fields such as magnetic field, thermal radiation, and chemical reactions, the stream associated with the transport characteristics of heat and mass can be substantially changed. The fluid’s motion is also altered by the velocity profiles as it is interacted by the magnetic lines due to the Lorentz forces. In addition, thermal radiation becomes more significant as the temperature increases, while the mass transfer and concentration profiles may be significantly altered because of chemical reactions like absorption or generation of species. In the following, few articles about the study of the stream associated with the transport of heat and mass across an expandable sheet are given, especially as they relate to micropolar fluids and their uses in polymer processing, material cooling, and biofluid dynamics.

In Aouadi^1^, the finite element method (FEM) and Chebyshev finite difference method were strongly used to solve the governing equations for the flow of micropolar fluid above an expandable sheet associated with the transport of heat and mass. Ishak et al.^2^ investigated the similarity solutions pertaining to stagnation-point flow over a shrinking sheet that is submerged in an incompressible micropolar fluid. Ishak^3^ investigated the evolution of the thermal boundary layer generated by a stretching sheet submerged in a micropolar fluid, considering the influence of thermal radiation. The influence of the transport of heat on the axisymmetric flow of magnetohydrodynamic micropolar fluid between two radially expandable sheets is detailed by Hayat et al.^4^. Mahmoud and Waheed^5^ conducted a study on the flow and the transport characteristics of heat of an electrically conducting micropolar fluid above a nonlinear expandable surface, considering variable wall heat flux, non-uniform heat generation/absorption, and a non-uniform transverse magnetic field. The study conducted by Das^6^ examined the impact of thermal radiation and chemical reactions on the unsteady magnetohydrodynamic free convection associated with the transport of heat and mass of a micropolar fluid flowing past a vertical porous plate within a rotating reference frame. Research conducted by Pal and Talukdar^7^ investigated the interplay between first-order chemical reactions and thermal radiation on the transport of heat and mass in the flow of micropolar fluids above a vertical porous plate. This study also considered the effects of heat generation and slip conditions at the porous boundary, alongside the influence of a transverse magnetic field. Mahmoud and Waheed^8^ conducted a study on the flow and the heat transport characteristics of an electrically conducting micropolar fluid over a permeable expandable surface. Their research considered variable heat flux, the influence of slip velocity on the surface, heat generation/absorption, and the effects of a uniform transverse magnetic field. Hussain et al.^9^ conducted a study on the thermal boundary layer flow of a micropolar fluid above an expandable sheet, considering the effects of radiation. This was achieved through a series of solutions derived using the homotopy analysis method (HAM). Sheri and Shamshuddin^10^ investigated the transport characteristics of heat and mass of magnetohydrodynamic micropolar fluid, considering the effects of viscous dissipation and chemical reactions. In their research, Mohanty et al.^11^ performed a numerical analysis of the mass transport characteristics of magnetohydrodynamic (MHD) unsteady mixed convection flow of a micropolar fluid originating from an expandable surface and traversing through a porous medium. The findings of Elbashbeshy et al.^12,13^ highlighted the essential role of thermal radiation and the unique characteristics of nanofluids in flow and transport processes above an expandable cylinder in a porous medium. They demonstrated that the unsteady flow of micropolar Maxwell fluids across an expandable surface is significantly affected by the presence of a magnetic field. The findings offer a deeper understanding of the velocity profiles, temperature distributions, and mass transport in MHD flows of viscoelastic fluids.

Fatunmbi and Samuel^14^ investigated the transport mechanism of temperature within a boundary layer of a magneto-micropolar fluid, characterized by temperature-dependent material properties, as it flows over a flat expandable sheet situated in a porous medium. Swain et al.^15^ examined the effects of viscous and Joule dissipation on magnetohydrodynamic (MHD) flow and gradient heat transfer over an expandable sheet situated within a porous medium. The 3D flow and heat transfer of a micropolar fluid containing a mixture of silver and copper oxide nanoparticles suspended in water driven by an exponentially stretching surface is effectively studied using numerical methods by Manjunatha et al.^16^. Dawar et al.^17^ performed a theoretical analysis of the transport properties of heat and mass of two-dimensional micropolar fluid flow, incorporating chemical reactions and drawing inspiration from the phenomena of Joule heating, thermophoresis, magnetic fields, and Brownian motion.

In Bilal et al.^18^, an innovative nonlinear diffusion model for magneto-micropolar fluids integrates Joule heating and velocity slip effects, yielding a more accurate portrayal of non-Newtonian fluid behavior than standard linear models. Yasir et al.^19^ explored the flow dynamics and thermal transport of hybrid nanofluids on an inclined shrinking surface, considering the influences of Ohmic heating, heat sources and sinks, and thermal radiation. Bilal et al.^20^ primarily analyzed the thermal and flow properties of a non-Newtonian micropolar nanofluid as it traverses a micropolar channel with porous walls under the influence of a changing magnetic field by exploring the hydrothermal behavior.

A new similarity transformation significantly impacted the analysis of Casson nanofluid flow and heat transfer over a nonlinear stretching sheet, particularly when considering nanoparticle shape effects and thermal radiation by Gireesha et al.^21^. The flow of an EMHD micropolar fluid through a microstructural slipped surface, incorporating a heat source/sink and chemical reactions, is analyzed by Ramesh et al.^22^. Also, Gireesha et al.^23^ investigated the enhanced heat transfer of a Casson hybrid nanofluid in blood through a stretching sheet, considering thermal radiation and various parameters. The investigation combined analytical and numerical methods to analyze the flow and heat transfer characteristics.

The study at hand develops a mathematical model to analyze the flow of micropolar fluid above an expandable sheet, influenced by a magnetic field, thermal radiation, and chemical reactions. The governing equations are reduced through suitable conversions, leading to a system of ordinary differential equations. This system is then numerically solved using the Legendre-collocation method, which is recognized for its precision and effectiveness in addressing boundary value problems, especially those involving non-linear equations. The focus of this research is to find scientific answers to the questions below.

1. How will the Legendre-collocation method be used and developed to obtain more accurate semi-analytical solution for an extrusion process problem?
2. What effects do the increasing of the magnetic field, thermal radiation, and chemical reaction have on the flow, heat, and mass transport during an extrusion process?
3. How will increasing the magnetic field, thermal radiation, and chemical reaction affect the quality of the extruded product via reducing the surface skin friction and increasing the surface heat flux?

Hence, the present study is motivated by the challenges associated with obtaining analytical solutions for the extrusion process problem and analyzing these solutions to enhance the quality of the extruded product. Therefore, the aim is to derive a semi-analytical solution that approximates the analytical one and improves the convergence of the results obtained. This is achieved through the application of the Legendre-collocation method, which highlights the innovative aspect of the current research.

## Mathematical formulation

**Fig. 1:**
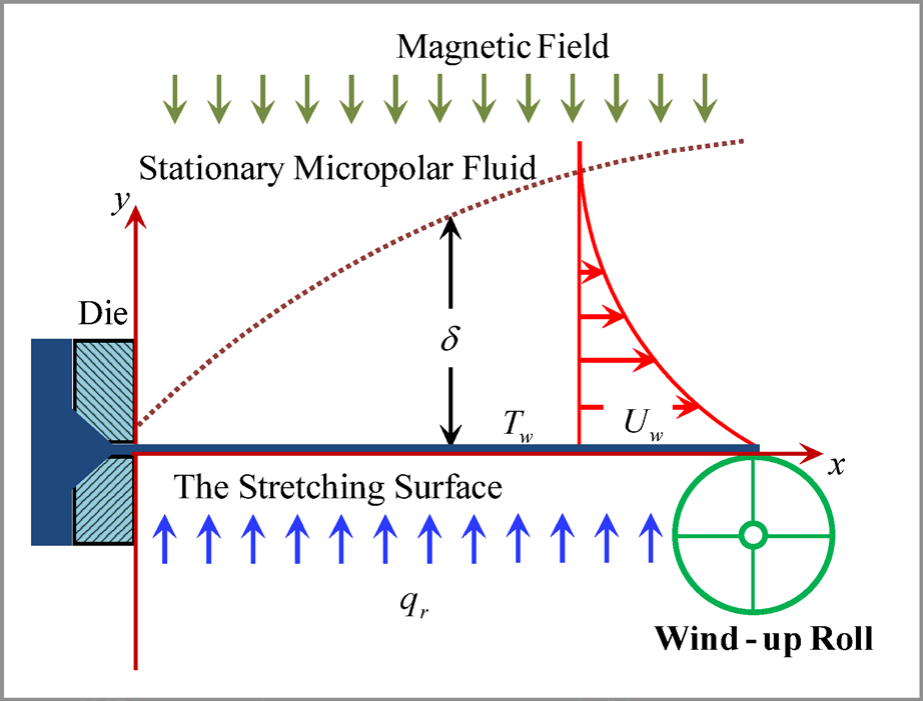
Physical model and coordinate systems.

Mathematical modeling is more explicitly tied to some physical assumptions as exhibited in Fig. 1. It contemplates a steady, laminar, free convection, and incompressible flow of a micropolar fluid above an expandable sheet $\:\left(y=0\right)$ in the existence of a magnetic field, thermal radiation, and chemical reaction. The fluid is electrically conductive while the excited electric field is zero and the Hall effect is ignored. A magnetic field of intensity $\:B$ is oriented normally to the surface, with the induced magnetic field being overlooked due to the assumption of a minimal magnetic Reynolds number. Also, a radiation heat flux $\:{q}_{r}$ and a chemical reaction of rate $\:{k}_{r}$ are all applied to the surface. The linear velocity $\:{U}_{w}\left(x\right)={U}_{0}x$ is expanding the surface, and the fluid is transported on $\:x-$axis while $\:y-$axis is normal to the surface axis. The temperature distribution of the surface is nonlinear with $\:{T}_{w}\left(x\right)={T}_{\infty\:}+{T}_{0}{x}^{2}$ where $\:{U}_{0}>0$ and $\:{T}_{0}>0$ are constants and $\:{T}_{\infty\:}$ is the ambient temperature.

The governing equations in dimensional form are^2^:

1 $$\:\frac{\partial\:u}{\partial\:x}+\frac{\partial\:w}{\partial\:y}=0,$$

2 $$\:u\frac{\partial\:u}{\partial\:x}+w\frac{\partial\:u}{\partial\:y}=\left(\nu\:+\frac{k}{\rho\:}\right)\frac{{\partial\:}^{2}u}{\partial\:{y}^{2}}+\frac{k}{\rho\:}\frac{\partial\:N}{\partial\:y}-\frac{\sigma\:{B}^{2}}{\rho\:}u,$$

3 $$\:u\frac{\partial\:N}{\partial\:x}+w\frac{\partial\:N}{\partial\:y}=\frac{\gamma\:}{\xi\:\rho\:}\frac{{\partial\:}^{2}N}{\partial\:{y}^{2}}-\frac{k}{J\rho\:}\left(2N+\frac{\partial\:u}{\partial\:y}\right),$$

4 $$\:u\frac{\partial\:T}{\partial\:x}+w\frac{\partial\:T}{\partial\:y}=\frac{\kappa\:}{\rho\:{c}_{p}}\frac{{\partial\:}^{2}T}{\partial\:{y}^{2}}+\frac{\mu\:+k}{\rho\:{c}_{p}}{\left(\frac{\partial\:u}{\partial\:y}\right)}^{2}+\frac{\sigma\:{B}^{2}}{\rho\:{c}_{p}}{u}^{2}-\frac{1}{\rho\:{c}_{p}}\frac{\partial\:{q}_{r}}{\partial\:y},$$

5 $$\:u\frac{\partial\:C}{\partial\:x}+w\frac{\partial\:C}{\partial\:y}=D\frac{{\partial\:}^{2}C}{\partial\:{y}^{2}}-{k}_{r}\left(C-{C}_{\infty\:}\right),$$

where $\:u$ and $\:w$ are the fluid velocity components in the $\:x$- and $\:y$-directions, respectively, $\:N$ is the fluid angular velocity, $\:T$ is the fluid temperature, $\:C$ is the fluid mass concentration, $\:\nu\:$, $\:k$, and $\:\mu\:$ are kinematic, vortex, and dynamic viscosities, respectively, $\:\sigma\:$ is the fluid electrical conductivity, $\:\rho\:$ is the fluid mass density, $\:{c}_{p}$ is the heat capacity at constant pressure, $\:\xi\:=v/{U}_{0}$ is the microinertia density, $\:\gamma\:$ is the spin gradient viscosity which is assumed to be $\:\left(\mu\:+k/2\right)\xi\:$, $\:\kappa\:$ is the fluid thermal conductivity, $\:D$ is the coefficient of the mass diffusivity, and $\:{C}_{\infty\:}$ is the ambient concentration. The term $\:\sigma\:{B}^{2}u/\rho\:$ represents the Lorentz force due to the interaction between the magnetic field and the fluid’s conductivity, the term $\:\left(\mu\:+k\right){\left(\partial\:u/\partial\:y\right)}^{2}/\rho\:{c}_{p}$ represents viscous dissipation, and the term $\:\sigma\:{B}^{2}{u}^{2}/\rho\:{c}_{p}$ indicates ohmic heating effect.

The governing conditions are:

6 $$\:u={U}_{w}\left(x\right),\:\:\:\:\:w=0,\:\:\:\:\:T={T}_{w}\left(x\right),\:\:\:\:\:N=-s\frac{\partial\:u}{\partial\:y},\:\:\:\:\:C={C}_{w},\:\:\:\:\:\text{a}\text{t}\:\:y=0,$$

7 $$\:\underset{y\to\:\infty\:}{\text{l}\text{i}\text{m}}\:u=\underset{y\to\:\infty\:}{\text{l}\text{i}\text{m}}\:N=0,\:\:\underset{y\to\:\infty\:}{\text{l}\text{i}\text{m}}\:T={T}_{\infty\:},\:\:\underset{y\to\:\infty\:}{\text{l}\text{i}\text{m}}\:C={C}_{\infty\:},$$.

where $\:{C}_{w}$ is the constant fluid mass concentration adjacent to the surface and $\:s$ is the surface parameter varying from $\:s=0$ (no slip condition, i.e., the fluid particles are neither rotating nor translating) to $\:s=1$.

Equation (1) represents the mass conservation. If the fluid is incompressible, the mass continuity equation is simplified to the given form which means that the divergence of the velocity field is zero everywhere. The momentum Eq. (2) describes how the momentum of a fluid changes due to forces acting on it. The convective transport of momentum is represented by $\:u\left(\partial\:u/\partial\:x\right)+w\left(\partial\:u/\partial\:y\right)$, and the right-hand side of Eq. (2) is consisting of the viscous force due to the fluid velocity and internal angular momentum and the Lorentz force. Equation (3) is needed to describe the conversation of internal angular momentum. The energy Eq. (4) describes the conservation of energy in a fluid. It accounts for the transport and transformation of energy due to conduction, convection, and heat transfer. The convective transport of temperature is $\:u\left(\partial\:T/\partial\:x\right)+w\left(\partial\:T/\partial\:r\right)$, and the right-hand side of Eq. (4) is describing the transport of heat due to viscous dissipation, ohmic heating, and a radiative heat flux $\:{q}_{r}$. Finally, the concentration Eq. (5) describes the conservation of mass concentration in a fluid. The convective transport of mass is $\:u\left(\partial\:C/\partial\:x\right)+w\left(\partial\:C/\partial\:r\right)$, and the right-hand side of Eq. (5) is describing the transport of mass due to mass diffusion and a chemical reaction of rate $\:{k}_{r}$.

From the boundary conditions given in Eqs. (6–7), the velocity/microrotation and temperature/concentration boundary layers are narrow zones that are close to a stretching surface, where the influence of viscosity, thermal, and mass diffusivity becomes prominent. Within these boundary layers, the fluid’s velocity/microrotation and temperature/concentration transition from Eq. (6) (due to the fact that the fluid layer is adjacent to the stretching surface) to Eq. (7) (because the fluid apart from the stretching surface is at rest and its temperature/concentration is ambient). The vertical component of the fluid’s velocity is kept at zero.

As per the Rosseland approximation^15^, the radiation heat flux is assumed to be

8 $$\:{q}_{r}=-\frac{4{\sigma\:}^{*}}{3{\alpha\:}^{*}}\frac{\partial\:{T}^{4}}{\partial\:y},$$.

where $\:{\sigma\:}^{*}$ is the Stefan-Boltzmann constant, $\:{\alpha\:}^{*}$ is the mean absorption coefficient, and $\:{T}^{4}$ is linearized by $\:4{T}_{\infty\:}^{3}T-3{T}_{\infty\:}^{4}$ after using Taylor’s series expansion about $\:{T}_{\infty\:}$.

To adapt the ruling scheme of partial differential equations, (1–3), into a scheme of ordinary differential equations, the similarity dimensionless variable

9 $$\:\eta\:=\sqrt{\frac{{U}_{w}}{\nu\:x}}\:y,$$.

is utilized. Through the following substitutions, the momentum/energy equation is simplified, and the continuity equation is satisfied:

10 $$\:u={U}_{w}{f}^{{\prime\:}}\left(\eta\:\right),\:\:w=-\sqrt{\frac{\nu\:{U}_{w}}{x}}f\left(\eta\:\right),\:\:N=\sqrt{\frac{{U}_{w}^{3}}{\nu\:x}}g\left(\eta\:\right),$$

11 $$\:T={T}_{\infty\:}\left(1+\left({T}_{R}-1\right)\theta\:\left(\eta\:\right)\right),\:\:C={C}_{\infty\:}\left(1+\left({C}_{R}-1\right)\varphi\:\left(\eta\:\right)\right),$$.

where $\:{T}_{R}={T}_{w}/{T}_{\infty\:}$ and $\:{C}_{R}={C}_{w}/{C}_{\infty\:}$ are the surface temperature excess and concentration excess ratios, and $\:f\left(\eta\:\right)$, $\:g\left(\eta\:\right)$, $\:\theta\:\left(\eta\:\right)$ and $\:\varphi\:\left(\eta\:\right)$ are the dimensionless stress, microrotation, temperature, and concentration functions, respectively. When Eqs. (9)-(11) are substituted into Eqs. (1)-(7), the mathematical model is reduced to a set of ordinary differential equations:

12 $$\:\left(1+R\right){f}^{{\prime\:}{\prime\:}{\prime\:}}+f{f}^{{\prime\:}{\prime\:}}-{\left({f}^{{\prime\:}}\right)}^{2}+R{g}^{{\prime\:}}-M{f}^{{\prime\:}}=0,$$

13 $$\:\left(1+\frac{R}{2}\right){g}^{{\prime\:}{\prime\:}}+f{g}^{{\prime\:}}-{f}^{{\prime\:}}g-R\left(2g+{f}^{{\prime\:}{\prime\:}}\right)=0,$$

14 $$\:\left(\frac{1+{N}_{R}}{\text{P}\text{r}}\right){\theta\:}^{{\prime\:}{\prime\:}}+f{\theta\:}^{{\prime\:}}-2{f}^{{\prime\:}}\theta\:+\text{E}\text{c}\:\left(\left(1+R\right){\left({f}^{{\prime\:}{\prime\:}}\right)}^{2}+M{\left({f}^{{\prime\:}}\right)}^{2}\right)=0,$$

15 $$\:\frac{1}{\text{S}\text{c}}{\varphi\:}^{{\prime\:}{\prime\:}}+f{\varphi\:}^{{\prime\:}}-{k}_{r}\varphi\:=0,$$

subjected to the boundary conditions:

16 $$\:f\left(0\right)=0,\:\:{f}^{{\prime\:}}\left(0\right)=1,\:\:g\left(0\right)=-s{f}^{{\prime\:}{\prime\:}}\left(0\right),\:\:\theta\:\left(0\right)=1,\:\:\varphi\:\left(0\right)=1,\:$$

17 $$\:\underset{\eta\:\to\:\infty\:}{\text{l}\text{i}\text{m}}\:{f}^{{\prime\:}}\left(\eta\:\right)=\underset{\eta\:\to\:\infty\:}{\text{l}\text{i}\text{m}}\:g\left(\eta\:\right)=\underset{\eta\:\to\:\infty\:}{\text{l}\text{i}\text{m}}\:\theta\:\left(\eta\:\right)=\underset{\eta\:\to\:\infty\:}{\text{l}\text{i}\text{m}}\:\varphi\:\left(\eta\:\right)=0,$$

where primes denote differentiation with respect to $\:\eta\:,$, $\:R=k/\mu\:$ is a material parameter, $\:M=\sigma\:{B}^{2}/\rho\:{U}_{0}$ is the magnetic parameter, $\:\text{P}\text{r}=\mu\:{c}_{p}/\kappa\:\:$ is the Prandtl number, $\:{N}_{R}=16{\sigma\:}^{*}{T}_{\infty\:}^{3}/3{\alpha\:}^{*}\kappa\:$ is the thermal radiation parameter, $\:\text{E}\text{c}={U}_{0}^{2}/{c}_{p}{T}_{0}$ is the Eckert number, and $\:\text{S}\text{c}=\nu\:/D$ is the Schmidt number.

The physical quantities of interest are the skin friction coefficient, $\:{C}_{f}$, the local Nusselt number, $\:{\text{N}\text{u}}_{x}$, the local Sherwood number, $\:{\text{S}\text{h}}_{x}$, and the dimensionless surface couple stress, $\:{M}_{x}$ defined as follows

18 $$\:{C}_{f}=\frac{2{\tau\:}_{w}}{\rho\:{U}_{w}^{2}},\:\:{\text{N}\text{u}}_{x}=\frac{x\left({q}_{w}+{q}_{r}\right)}{\kappa\:\left({T}_{w}-{T}_{\infty\:}\right)},\:\:{\text{S}\text{h}}_{x}=\frac{x{q}_{m}}{D\left({C}_{w}-{C}_{\infty\:}\right)},\:\:{M}_{x}=\frac{{m}_{w}}{\rho\:x{U}_{w}^{2}},$$.

where $\:{\tau\:}_{w}=-{\left[\left(\mu\:+k\right)\left(\partial\:u/\partial\:y\right)\:+kN\right]}_{y=0}$ is the surface shear stress, $\:{q}_{w}=-\kappa\:{\left(\partial\:T/\partial\:y\right)}_{y=0}$ is the surface heat flux, $\:{q}_{m}=-D{\left(\partial\:C/\partial\:y\right)}_{y=0}$ is the surface mass flux, and $\:{m}_{w}=\gamma\:{\left(\partial\:N/\partial\:y\right)}_{y=0}$ is the surface couple stress. The modified skin friction coefficient, the modified Nusselt number, the modified Sherwood number, and the modified surface couple stress are obtained by substituting the dimensionless similarity transformations (9–11) into Eq. (18) and are defined as follows:

19 $$\:\frac{1}{2}{C}_{f}\sqrt{{\text{R}\text{e}}_{x}}=-\left(1+\left(1-s\right)R\right){f}^{{\prime\:}{\prime\:}}\left(0\right),\:\:\frac{{\text{N}\text{u}}_{x}}{\sqrt{{\text{R}\text{e}}_{x}}}=-\left(1+{N}_{R}\right){\theta\:}^{{\prime\:}}\left(0\right),\:$$

20 $$\:\frac{{\text{S}\text{h}}_{x}}{\sqrt{{\text{R}\text{e}}_{x}}}=-{\varphi\:}^{{\prime\:}}\left(0\right),\:\:{M}_{x}{\text{R}\text{e}}_{x}\:=-\left(1+\frac{R}{2}\right){g}^{{\prime\:}}\left(0\right),$$.

where $\:{\text{R}\text{e}}_{x}=x{U}_{w}/\nu\:$ is the local Reynolds number.

### Numerical method

When the Legendre differential equation is solved, $\:{P}_{n}\left(\eta\:\right)$, the first type Legendre polynomial of rank $\:n$, is generated. It’s described by^24^

21 $$\:{P}_{i}\left(t\right)=\frac{1}{{2}^{i}}\displaystyle\sum\:_{l=0}^{\lfloor\frac{i}{2}\rfloor}{\left(-1\right)}^{l}\left(\begin{array}{c}i\\\:l\end{array}\right)\left(\begin{array}{c}2i-2l\\\:i\end{array}\right){t}^{i-2l},$$.

where $\:t\in\:\left[-1,\:1\right]$. To map the interval of $\:t$ from $\:\left[-1,\:1\right]$ to $\:\left[0,\:1\right]$, the shifted Legendre polynomials, $\:{\stackrel{\sim}{P}}_{i}\left(t\right)$, is used where $\:{\stackrel{\sim}{P}}_{i}\left(t\right)={P}_{i}\left(2t-1\right)$ and

22 $$\:\left(i+1\right){\stackrel{\sim}{P}}_{i+1}\left(t\right)=\left(2i+1\right)\left(2t-1\right){\stackrel{\sim}{P}}_{i}\left(t\right)-i{\stackrel{\sim}{P}}_{i-1}\left(t\right),\:\:i=1,\:2,\dots\:\:.$$.

Referred to the detailed explanation presented in^24,25^, consider that a function $\:{y}_{k}\left(t\right)\in\:{L}^{2}\left[\text{0,1}\right]$ is approximated by

23 $$\:{y}_{k}\left(t\right)\cong\:\sum\:_{i=0}^{n}{c}_{ik}{\stackrel{\sim}{P}}_{i}\left(t\right)=\:\mathbf{P}\left(t\right)\:{\mathbf{C}}_{k},$$

where $\:{L}^{2}\left[\text{0,1}\right]\:$is the set of all square integrable function defined over the interval $\:\left[\text{0,1}\right]$, $\:{c}_{ik}$ is unknown coefficient required to determine $\:{y}_{k}\left(t\right)$, $\:\mathbf{P}\left(t\right)=\left[{\stackrel{\sim}{P}}_{0}\left(t\right)\:\:{\stackrel{\sim}{P}}_{1}\left(t\right)\cdots\:{\stackrel{\sim}{P}}_{n}\left(t\right)\right],\:$and $\:{\mathbf{C}}_{k}=\:{\left[{c}_{0k}\:{c}_{1k}\cdots\:{c}_{nk}\right]}^{T}$. Also, based on Eq. (23), it can be said that.

24 $$\:\frac{{d}^{\alpha\:}{y}_{k}\left(t\right)}{d{t}^{\alpha\:}}={y}_{k}^{\left(\alpha\:\right)}\left(t\right)\cong\:\sum\:_{i=0}^{n}{c}_{ik}{\stackrel{\sim}{P}}_{i}^{\left(\alpha\:\right)}\left(t\right)=\:\mathbf{P}\left(t\right){\:\left({\mathbf{D}}^{\alpha\:}\right)}^{T}{\mathbf{C}}_{k},\:\:\alpha\:\in\:{\mathbb{Z}}^{+}$$

25 $$\:{\left({y}_{k}^{\left(1\right)}\left(t\right)\right)}^{2}\cong\:\sum\:_{i=0}^{n}\sum\:_{j=0}^{n}{c}_{ik}{c}_{jk}{\stackrel{\sim}{P}}_{i}^{\left(1\right)}\left(t\right){\stackrel{\sim}{P}}_{j}^{\left(1\right)}\left(t\right)=\:\mathbf{P}\left(t\right)\:\mathbf{D}^{T}\:{\mathbf{P}}^{\varvec{*}}\left(t\right){{\mathbf{D}}^{\mathbf{*}}}{\mathbf{C}}_{k}^{*},$$

26 $$\:{\left({y}_{k}^{\left(2\right)}\left(t\right)\right)}^{2}\cong\:\sum\:_{i=0}^{n}\sum\:_{j=0}^{n}{c}_{ik}{c}_{jk}{\stackrel{\sim}{P}}_{i}^{\left(2\right)}\left(t\right){\stackrel{\sim}{P}}_{j}^{\left(2\right)}\left(t\right)=\:\mathbf{P}\left(t\right)\:\left({\mathbf{D}}^{2}\right)^{T}\:{\mathbf{P}}^{\varvec{*}}\left(t\right){\left({{\mathbf{D}}^{\mathbf{*}}}\right)}^{2}{\mathbf{C}}_{k}^{*},$$

27 $$\:{y}_{k}\left(t\right){y}_{k}^{\left(1\right)}\left(t\right)\cong\:\sum\:_{i=0}^{n}\sum\:_{j=0}^{n}{c}_{ik}{c}_{jk}{\stackrel{\sim}{P}}_{i}\left(t\right){\stackrel{\sim}{P}}_{j}^{\left(1\right)}\left(t\right)=\mathbf{P}\left(t\right){\mathbf{P}}^{\varvec{*}}\left(t\right){\mathbf{D}}^{\mathbf{*}}{\mathbf{C}}_{k}^{*},$$

28 $$\:{y}_{k}\left(t\right){y}_{k}^{\left(2\right)}\left(t\right)\cong\:\sum\:_{i=0}^{n}\sum\:_{j=0}^{n}{c}_{ik}{c}_{jk}{\stackrel{\sim}{P}}_{i}\left(t\right){\stackrel{\sim}{P}}_{j}^{\left(2\right)}\left(t\right)=\mathbf{P}\left(t\right){\mathbf{P}}^{\varvec{*}}\left(t\right){\left({{\mathbf{D}}^{\mathbf{*}}}\right)}^{2}{\mathbf{C}}_{k}^{*},$$

where $\:{\mathbf{C}}_{k}^{*}={\left[{c}_{0k}^{2},\:{c}_{0k}{c}_{1k},\cdots\:,{c}_{0k}{c}_{nk}\cdots\:,{c}_{nk}{c}_{nk}\right]}^{T}$ and $\:\mathbf{D}$ is the operating derivative square matrix defined in^24,25^ and its size is $\:(n+1)$. Also, the matrices $\:{\mathbf{P}}^{\text{*}}\left(t\right)$ and $\:{\mathbf{D}}^{\text{*}}\left(t\right)$ are assembled as follows.

$$\:{\mathbf{P}}^{{*}}\left(t\right)={\left[\begin{array}{ccc}\mathbf{P}\left(t\right)&\:\cdots\:&\:\mathbf{O}\\\:\vdots&\:\ddots\:&\:\vdots\\\:\mathbf{O}&\:\cdots\:&\:\mathbf{P}\left(t\right)\end{array}\right]}_{(n+1)\times\:{(n+1)}^{2}},\:\:{\mathbf{D}}^{\text{*}}\left(t\right)={\left[\begin{array}{ccc}\mathbf{D}^{T}&\:\cdots\:&\:\mathbf{O}\\\:\vdots&\:\ddots\:&\:\vdots\\\:\mathbf{O}&\:\cdots\:&\:\mathbf{D}^{T}\end{array}\right]}_{(n+1)\times\:{(n+1)}^{2}}.$$

Now, the problem given in the system of Eqs. (12)-(17)is solved asymptotically using the Legendre-collocation method. At the beginning, the domain of solution is changed from $\:[0,{\eta\:}_{\infty\:}\:]$ to $\:\left[0,\:1\right]$ by using the linear transformation $\:\eta\:={\eta\:}_{\infty\:}t$. By assuming $\:f={y}_{1}$, $\:g={y}_{2}$, $\:\theta\:={y}_{3}$, and $\:\varphi\:={y}_{4}$, the new format of the problem system is as follows:

29 $$\:\frac{1}{{\eta\:}_{\infty\:}^{2}}\left(1+R\right){y}_{1}^{\left(3\right)}+\frac{1}{{\eta\:}_{\infty\:}}{y}_{1}{y}_{1}^{\left(2\right)}-\frac{1}{{\eta\:}_{\infty\:}}{\left({y}_{1}^{\left(1\right)}\right)}^{2}+R{y}_{2}^{\left(1\right)}-M{y}_{1}^{\left(1\right)}=0,$$

30 $$\:\frac{1}{{\eta\:}_{\infty\:}^{2}}\left(1+\frac{R}{2}\right){y}_{2}^{\left(2\right)}+\frac{1}{{\eta\:}_{\infty\:}}{y}_{1}{y}_{2}^{\left(1\right)}-\frac{1}{{\eta\:}_{\infty\:}}{y}_{1}^{\left(1\right)}{y}_{2}-R\left(2{y}_{2}+\frac{1}{{\eta\:}_{\infty\:}^{2}}{y}_{1}^{\left(2\right)}\right)=0,$$

31 $$\:\frac{1}{{\eta\:}_{\infty\:}}\left(\frac{1+{N}_{R}}{\text{P}\text{r}}\right){y}_{3}^{\left(2\right)}+{y}_{1}{y}_{3}^{\left(1\right)}-2{y}_{1}^{\left(1\right)}{y}_{3}+\text{E}\text{c}\:\left(\frac{1}{{\eta\:}_{\infty\:}^{3}}\left(1+R\right){\left({y}_{1}^{\left(2\right)}\right)}^{2}+\frac{M}{{\eta\:}_{\infty\:}}{\left({y}_{1}^{\left(1\right)}\right)}^{2}\right)=0,$$

32 $$\:\frac{1}{\text{S}\text{c}\:{\eta\:}_{\infty\:}^{2}}{y}_{4}^{\left(2\right)}+\frac{1}{{\eta\:}_{\infty\:}}{y}_{1}{y}_{4}^{\left(1\right)}-{k}_{r}{y}_{4}=0,$$

subjected to the boundary conditions:

33 $$\:{y}_{1}\left(0\right)=0,\:\:{y}_{1}^{\left(1\right)}\left(0\right)={\eta\:}_{\infty\:},\:\:{y}_{2}\left(0\right)=-\frac{s}{{\eta\:}_{\infty\:}^{2}}\:{y}_{1}^{\left(2\right)}\left(0\right),\:\:{y}_{3}\left(0\right)=1,\:\:{y}_{4}\left(0\right)=1,\:$$

34 $$\:\:{y}_{1}^{\left(1\right)}\left(1\right)={y}_{2}\left(1\right)=\:{y}_{3}\left(1\right)={y}_{4}\left(1\right)=0.$$

By recalling Eqs. (23)-(28) and substituting them into (29)-(34) then applying the collocation method using the nodes

35 $$\:{t}_{i}=\frac{1}{2}\left(\text{cos}\left(\frac{\left(n-i\right)\pi\:}{n}\right)\right),\:\:i=0,\:1,\:2,\:\dots\:,n\:,\:\:0\le\:t\le\:1,$$.

the new form of Eqs. (29)-(34) is as follows:

36 $$\:\mathbf{P}\left({t}_{i}\right)\left[\left(\frac{1+R}{{\eta\:}_{\infty\:}^{2}}\:{\:\left({\mathbf{D}}^{3}\right)}^{T}-M{\mathbf{D}}^{T}\right){\mathbf{C}}_{1}+R\:{\mathbf{D}}^{T}{\mathbf{C}}_{2}+\frac{1}{{\eta\:}_{\infty\:}}\left({\mathbf{P}}^{\varvec{*}}\left({t}_{i}\right){\left({{\mathbf{D}}^{\mathbf{*}}}\right)}^{2}-\:\:\mathbf{D}^{T}\:{\mathbf{P}}^{\varvec{*}}\left({t}_{i}\right){{\mathbf{D}}^{\mathbf{*}}}\right){\mathbf{C}}_{11}\right]=0,$$

37 $$\:\mathbf{P}\left({t}_{i}\right)\left[\left(\frac{1}{{\eta\:}_{\infty\:}^{2}}\left(1+\frac{R}{2}\right){\left({\mathbf{D}}^{2}\right)}^{T}-2R\:\mathbf{I}\right){\mathbf{C}}_{2}-\frac{R}{{\eta\:}_{\infty\:}^{2}}{\left({\mathbf{D}}^{2}\right)}^{T}{\mathbf{C}}_{1}+\frac{1}{{\eta\:}_{\infty\:}}{\mathbf{P}}^{\varvec{*}}\left({t}_{i}\right){\mathbf{D}}^{\mathbf{*}}\left({\mathbf{C}}_{21}-{\mathbf{C}}_{12}\right)\right]=0,$$

38 $$\begin{aligned} & \:\mathbf{P}\left({t}_{i}\right)\left[\frac{1}{{\eta\:}_{\infty\:}}\left(\frac{1+{N}_{R}}{\text{P}\text{r}}\right){\left({\mathbf{D}}^{2}\right)}^{T}{\mathbf{C}}_{3}+{\mathbf{P}}^{\varvec{*}}\left({t}_{i}\right){\mathbf{D}}^{\mathbf{*}}\left({\mathbf{C}}_{31}-2{\mathbf{C}}_{13}\right)\right.\\&\left.+\text{E}\text{c}\:{\mathbf{D}}^{T}\left(\frac{1}{{\eta\:}_{\infty\:}^{3}}\left(1+R\right)\:{\mathbf{D}}^{T}\:{\mathbf{P}}^{\varvec{*}}\left({t}_{i}\right){\mathbf{D}}^{\mathbf{*}}+\frac{M}{{\eta\:}_{\infty\:}}\:{\mathbf{P}}^{\varvec{*}}\left({t}_{i}\right)\right){\mathbf{D}}^{\mathbf{*}}{\mathbf{C}}_{11}\right]=0,\end{aligned}$$

39 $$\:\mathbf{P}\left({t}_{i}\right)\left[\left(\frac{1}{\text{S}\text{c}\:{\eta\:}_{\infty\:}^{2}}{\left({\mathbf{D}}^{2}\right)}^{T}-{k}_{r}\mathbf{I}\right){\mathbf{C}}_{4}+\frac{1}{{\eta\:}_{\infty\:}}{\mathbf{P}}^{\varvec{*}}\left({t}_{i}\right){{\mathbf{D}}^{\mathbf{*}}}{\mathbf{C}}_{41}\right]=0,$$

subjected to the boundary conditions:

40 $$\:\mathbf{P}\left(0\right){\mathbf{C}}_{1}=0,\:\:\:\mathbf{P}\left(0\right){\mathbf{D}}^{T}{\mathbf{C}}_{1}={\eta\:}_{\infty\:},\:\:\:\mathbf{P}\left(0\right)\left[{\mathbf{C}}_{2}+\frac{s}{{\eta\:}_{\infty\:}^{2}}{\:\left({\mathbf{D}}^{2}\right)}^{T}\right]{\mathbf{C}}_{1}=0,\:\:\:\mathbf{P}\left(0\right){\mathbf{C}}_{3}=\mathbf{P}\left(0\right){\mathbf{C}}_{4}=1,$$

41 $$\:\:\mathbf{P}\left(1\right){\mathbf{D}}^{T}{\mathbf{C}}_{1}=\:\mathbf{P}\left(1\right){\mathbf{C}}_{2}=\:\mathbf{P}\left(1\right){\mathbf{C}}_{3}=\:\mathbf{P}\left(1\right){\mathbf{C}}_{4}=0,$$

where $\:{\mathbf{C}}_{ij}$ is the tensor product of $\:{\mathbf{C}}_{i}$ and $\:{\mathbf{C}}_{j}$. The matrix form of the system (36)-(41) is

42 $$\:\mathbf{A}\mathbf{C}+\mathbf{B}\:\stackrel{\sim}{\mathbf{C}}+\mathbf{H}\:\stackrel{-}{\mathbf{C}}=\mathbf{F},$$.

where

$$\:\mathbf{A}=\left[\begin{array}{cccc}{\mathbf{A}}_{11}&\:{\mathbf{A}}_{12}&\:\mathbf{O}&\:\mathbf{O}\\\:{\mathbf{A}}_{21}&\:{\mathbf{A}}_{22}&\:\mathbf{O}&\:\mathbf{O}\\\:\mathbf{O}&\:\mathbf{O}&\:{\mathbf{A}}_{33}&\:\mathbf{O}\\\:\mathbf{O}&\:\mathbf{O}&\:\mathbf{O}&\:{\mathbf{A}}_{44}\end{array}\right],\:\:\mathbf{B}=\left[\begin{array}{cccc}{\mathbf{B}}_{11}&\:\mathbf{O}&\:\mathbf{O}&\:\mathbf{O}\\\:\mathbf{O}\:\:&\:\mathbf{O}&\:\mathbf{O}&\:\mathbf{O}\\\:{\mathbf{B}}_{31}&\:\mathbf{O}&\:\mathbf{O}&\:\mathbf{O}\\\:\mathbf{O}\:\:&\:\mathbf{O}&\:\mathbf{O}&\:\mathbf{O}\end{array}\right],\:\:\mathbf{H}=\left[\begin{array}{ccccc}\mathbf{O}&\:\mathbf{O}&\:\mathbf{O}&\:\mathbf{O}&\:\mathbf{O}\\\:{\mathbf{H}}_{21}&\:{\mathbf{H}}_{22}&\:\mathbf{O}&\:\mathbf{O}&\:\mathbf{O}\\\:\mathbf{O}&\:\mathbf{O}&\:{\mathbf{H}}_{33}&\:{\mathbf{H}}_{34}&\:\mathbf{O}\\\:\mathbf{O}&\:\mathbf{O}&\:\mathbf{O}&\:\mathbf{O}&\:{\mathbf{H}}_{45}\end{array}\right],$$

$$\:\mathbf{C}={\left[{\mathbf{C}}_{1}\:{\mathbf{C}}_{2}\:{\mathbf{C}}_{3}\:{\mathbf{C}}_{4\:}\right]}^{T},\:\:\stackrel{\sim}{\mathbf{C}}={\left[{\mathbf{C}}_{11}\:\mathbf{O}\:\mathbf{O}\:\mathbf{O}\right]}^{T},\:\:\stackrel{-}{\mathbf{C}}={\left[{\mathbf{C}}_{12}\:{\mathbf{C}}_{21}\:{\mathbf{C}}_{13}\:{\mathbf{C}}_{31}\:{\mathbf{C}}_{41}\right]}^{T},\:\:\mathbf{F}={\left[{\mathbf{F}}_{1}\:{\mathbf{O}}\:{\mathbf{F}}_{3}\:{\mathbf{F}}_{4}\right]}^{T}$$

$$\:{\mathbf{F}}_{1}={\left[\mathbf{O}\:\:|\:\:0,{\eta\:}_{\infty\:},0\right]}^{T}\:\:\:,\:\:{\mathbf{F}}_{3}={\mathbf{F}}_{4}={\left[\mathbf{O}\:\:|\:\:1,\:0\right]}^{T},\:\:{\mathbf{E}}_{1}=\frac{1}{{\eta\:}_{\infty\:}^{2}}\left(\left(1+R\right){\left({\mathbf{D}}^{3}\right)}^{T}-M{\:\mathbf{D}}^{T}\right),$$

$$\:{\mathbf{A}}_{11}={\left[\mathbf{P}\left({t}_{2}\right){\mathbf{E}}_{1},\:\dots\:,\mathbf{P}\left({t}_{n-1}\right){\mathbf{E}}_{1}\:\:|\:\:\:\mathbf{P}\left(0\right),\mathbf{P}\left(0\right){\mathbf{D}}^{T},\frac{s}{{\eta\:}_{\infty\:}^{2}}\mathbf{P}\left(0\right){\left({\mathbf{D}}^{2}\right)}^{T}\:\right]}^{T},\:\:{\mathbf{E}}_{2}=\frac{1}{{\eta\:}_{\infty\:}^{2}}\left(1+\frac{R}{2}\right){\left({\mathbf{D}}^{2}\right)}^{T}-2R\:\mathbf{I},$$

$$\:{\mathbf{A}}_{12}={\left[R\:\mathbf{P}\left({t}_{2}\right){\mathbf{D}}^{T},\:\dots\:,R\:\mathbf{P}\left({t}_{n-1}\right){\mathbf{D}}^{T}\:\:|\:\:\:0,0,\mathbf{P}\left(0\right)\:\right]}^{T},\:\:{\mathbf{A}}_{22}={\left[\mathbf{P}\left({t}_{1}\right){\mathbf{E}}_{2},\:\dots\:,\mathbf{P}\left({t}_{n-1}\right){\mathbf{E}}_{2}\:|\:\mathbf{P}\left(0\right),\mathbf{P}\left(1\right)\:\right]}^{T},$$

$$\:{\mathbf{A}}_{21}={\left[-\frac{R}{{\eta\:}_{\infty\:}^{2}}\mathbf{P}\left({t}_{1}\right){\left({\mathbf{D}}^{2}\right)}^{T},\:\dots\:,-\frac{R}{{\eta\:}_{\infty\:}^{2}}\mathbf{P}\left({t}_{n-1}\right){\left({\mathbf{D}}^{2}\right)}^{T}\:|\:\frac{s}{{\eta\:}_{\infty\:}^{2}}\mathbf{P}\left(0\right){\:\left({\mathbf{D}}^{2}\right)}^{T},\mathbf{P}\left(1\right){\:\mathbf{D}}^{T}\:\right]}^{T},$$

$$\:{\mathbf{A}}_{33}={\left[\mathbf{P}\left({t}_{1}\right)\left(\frac{1+{N}_{R}}{{\eta\:}_{\infty\:}\text{P}\text{r}}\right){\left({\mathbf{D}}^{2}\right)}^{T},\:\dots\:,\:\mathbf{P}\left({t}_{n-1}\right)\left(\frac{1+{N}_{R}}{{\eta\:}_{\infty\:}\text{P}\text{r}}\right){\left({\mathbf{D}}^{2}\right)}^{T}\:\:|\:\:\mathbf{P}\left(0\right),\:\mathbf{P}\left(1\right)\:\right]}^{T},$$

$$\:{\mathbf{A}}_{44}={\left[\mathbf{P}\left({t}_{1}\right){\mathbf{E}}_{4},\:\dots\:,\:\mathbf{P}\left({t}_{n-1}\right){\mathbf{E}}_{4}\:\:|\:\mathbf{P}\left(0\right),\:\mathbf{P}\left(1\right)\:\right]}^{T},\:\:{\mathbf{E}}_{4}=\frac{1}{\text{S}\text{c}\:{\eta\:}_{\infty\:}^{2}}{\left({\mathbf{D}}^{2}\right)}^{T}-{k}_{r}\mathbf{I},$$

$$\:{\mathbf{B}}_{11}=\frac{1}{{\eta\:}_{\infty\:}}\left[\begin{array}{c}P\left({t}_{2}\right)\left({\mathbf{P}}^{\varvec{*}}\left({t}_{2}\right){\left({{\mathbf{D}}^{\mathbf{*}}}\right)}^{2}-\:\:\mathbf{D}^{T}\:{\mathbf{P}}^{\varvec{*}}\left({t}_{2}\right){\mathbf{D}}^{\mathbf{*}}\right)\\\:\vdots\\\:P\left({t}_{n-1}\right)\left({\mathbf{P}}^{\varvec{*}}\left({t}_{n-1}\right){\left({{\mathbf{D}}^{\mathbf{*}}}\right)}^{2}-\:\:\mathbf{D}\:{\mathbf{P}}^{\varvec{*}}\left({t}_{n-1}\right){\mathbf{D}}^{\mathbf{*}}\right)\\\:\mathbf{O}\end{array}\right],\:\:$$

$$\:{\mathbf{H}}_{34}=-\frac{1}{2}{\mathbf{H}}_{33}=\left[\mathbf{P}\left({t}_{1}\right){\mathbf{P}}^{\varvec{*}}\left({t}_{1}\right){{\mathbf{D}}^{\mathbf{*}}},\dots\:,\:\mathbf{P}\left({t}_{n-1}\right){\mathbf{P}}^{\varvec{*}}\left({t}_{n-1}\right){{\mathbf{D}}^{\mathbf{*}}}\:|\:\mathbf{O}\:\right],$$

$$\:{\mathbf{H}}_{22}=-{\mathbf{H}}_{21}={\mathbf{H}}_{45}=\frac{1}{{\eta\:}_{\infty\:}}{\mathbf{H}}_{34},$$

$$\:{\mathbf{B}}_{31}=\text{E}\text{c}\:\left[\begin{array}{c}P\left({t}_{1}\right){\mathbf{D}^{T}}\left(\frac{1}{{\eta\:}_{\infty\:}^{3}}\left(1+R\right)\:{\mathbf{D}^{T}}{\mathbf{P}}^{\varvec{*}}\left({t}_{1}\right){{{\mathbf{D}}^{\mathbf{*}}}}+\frac{M}{{\eta\:}_{\infty\:}}{\mathbf{P}}^{\varvec{*}}\left({t}_{1}\right)\right){\mathbf{D^{*}}}\\\:\vdots\\\:P\left({t}_{n-1}\right){\mathbf{D}^{T}}\left(\frac{1}{{\eta\:}_{\infty\:}^{3}}\left(1+R\right)\:{\mathbf{D}}^{T}{\mathbf{P}}^{\varvec{*}}\left({t}_{n-1}\right){{{\mathbf{D}}^{\mathbf{*}}}}+\frac{M}{{\eta\:}_{\infty\:}}{\mathbf{P}}^{\varvec{*}}\left({t}_{n-1}\right)\right){\mathbf{D}}^{\mathbf{*}}\\\:\mathbf{O}\end{array}\right],$$.

and $\:\mathbf{O}$ is a zero matrix with adjustable dimensions based on its containing matrix. Additionally, the nonlinear system (42) has $\:4(n+1)$ unknowns. Newton technique is used to solve this system. Upon solving the system, the functions $\:f$, $\:g$, $\:\theta\:$, and $\:\varphi\:$ are generated after identifying the unknowns $\:{\mathbf{C}}_{1}$, $\:{\mathbf{C}}_{2}$, $\:{\mathbf{C}}_{3}$, and $\:{\mathbf{C}}_{4}$. Finally, the solutions of the issue are found via the inverse transformation $\:t=\eta\:/{\eta\:}_{\infty\:}$.

## Results and Discussions

For the developed algorithm of the pre mentioned numerical method, the CPU running time is different according to the value of $\:n$ be used. As the value of $\:n$ is increased, the accuracy of results will be increased. The CPU running times of the developed algorithm by using Mathematica software are $\:3$, $\:24$, $\:103$, and $\:425$ seconds for $\:n=5$, $\:10$, $\:15$, and $\:22$, respectively, after using $\:{\eta\:}_{\infty\:}=40$.

**Fig. 2:**
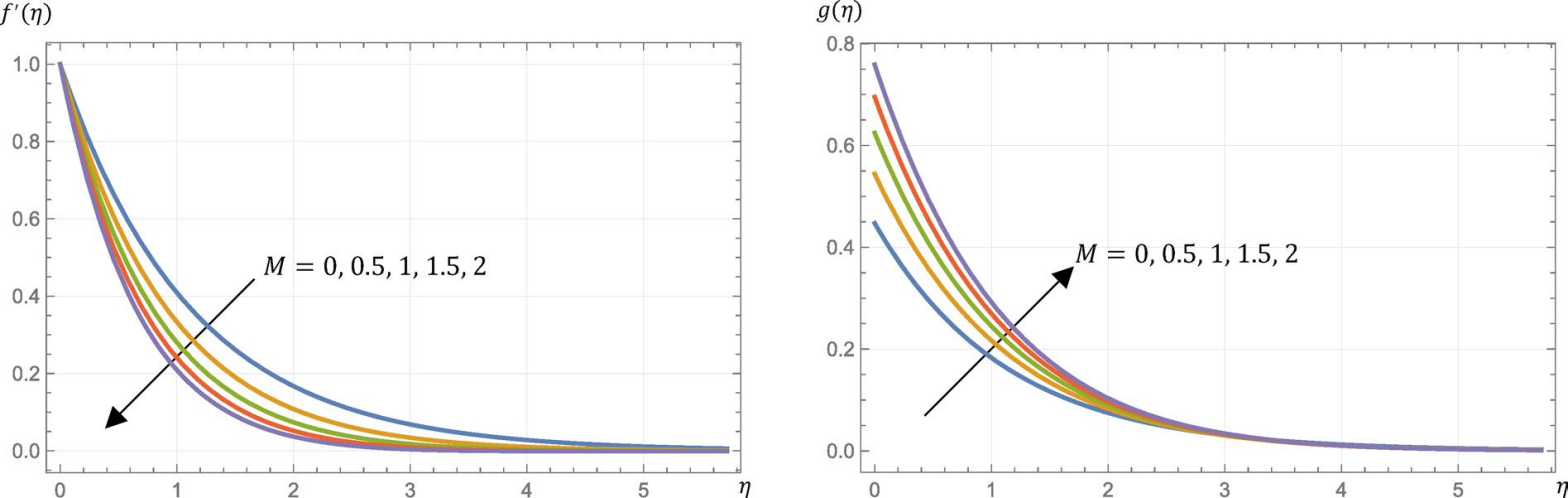
Micropolar fluid velocity, $\:{f}^{{\prime\:}}\left(\eta\:\right)$, and microrotation, $\:g\left(\eta\:\right)$, profiles at different values of the magnetic parameter, $\:M$, given that $\:R=0.5$, $\:\text{P}\text{r}=0.7$, $\:\text{E}\text{c}=0.2$, $\:\text{S}\text{c}=\:0.22$, $\:s=0.5$, $\:{N}_{R}=3$, and $\:{k}_{r}=0.5$.

**Fig. 3:**
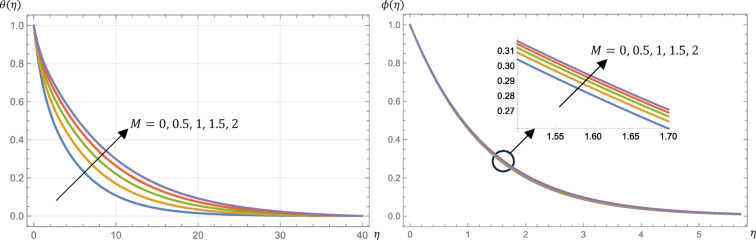
Micropolar fluid temperature, $\:\theta\:\left(\eta\:\right)$, and concentration, $\:\varphi\:\left(\eta\:\right)$, profiles at different values of the magnetic parameter, $\:M$, given that $\:R=0.5$, $\:\text{P}\text{r}=0.7$, $\:\text{E}\text{c}=0.2$, $\:\text{S}\text{c}=\:0.22$, $\:s=0.5$, $\:{N}_{R}=3$, and $\:{k}_{r}=0.5$.

**Fig. 4:**
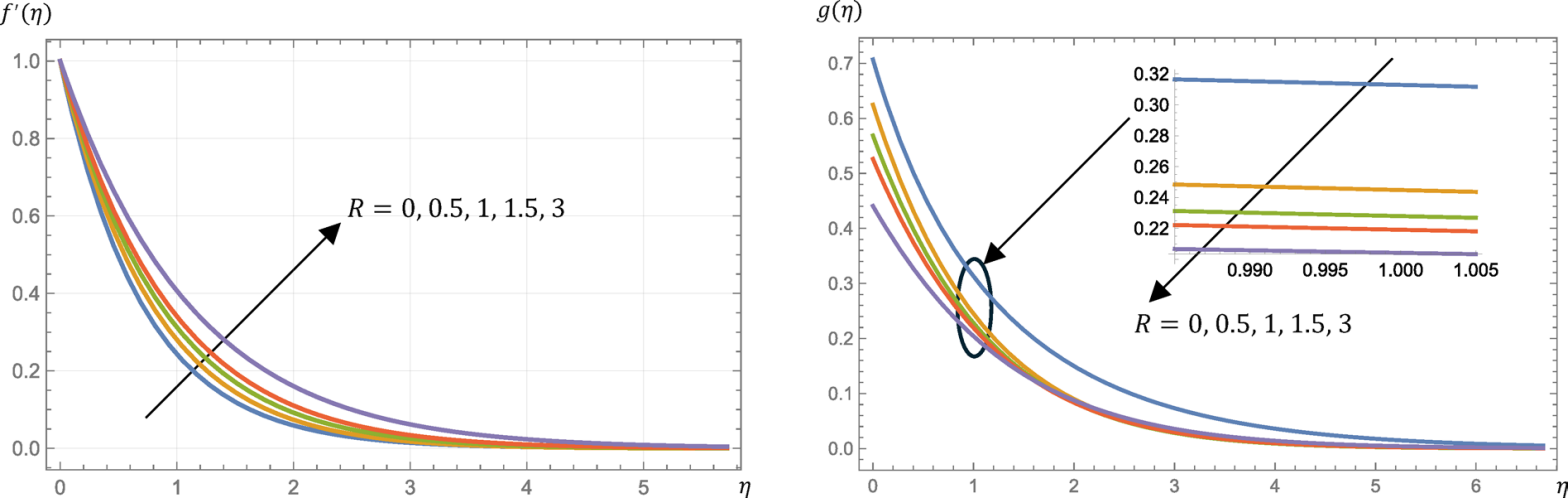
Micropolar fluid velocity, $\:{f}^{{\prime\:}}\left(\eta\:\right)$, and microrotation, $\:g\left(\eta\:\right)$, profiles at different values of the material parameter, $\:R$, given that $\:M=1$, $\:\text{P}\text{r}=0.7$, $\:\text{E}\text{c}=0.2$, $\:\text{S}\text{c}=\:0.22$, $\:s=0.5$, $\:{N}_{R}=3$, and $\:{k}_{r}=0.5$.

**Fig. 5:**
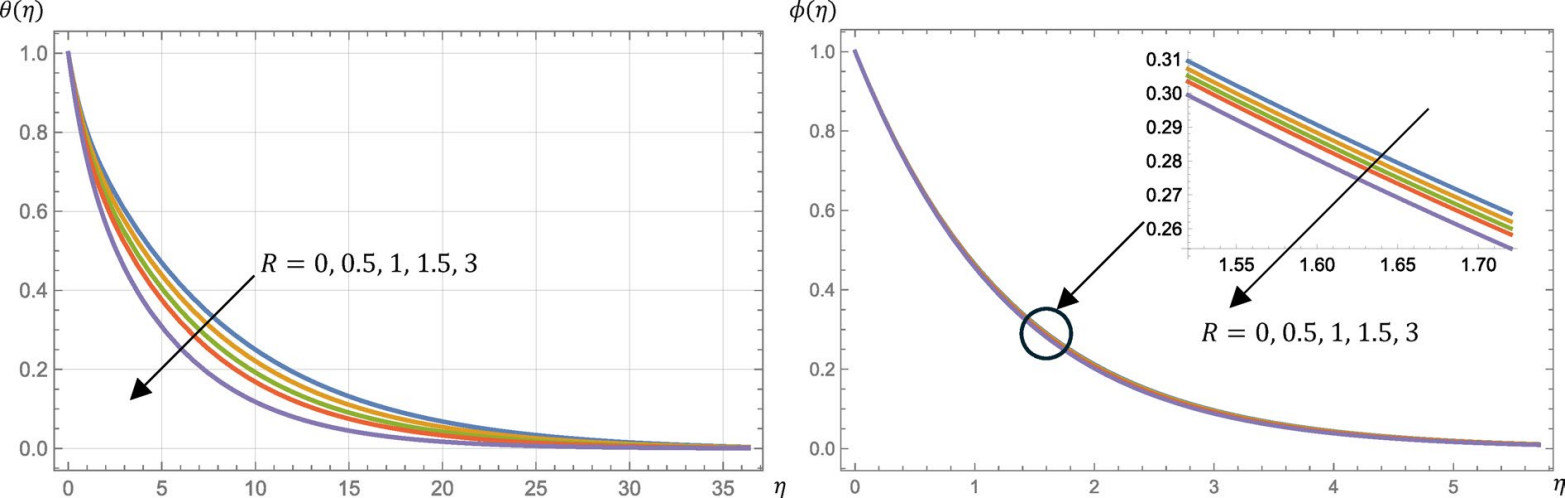
Micropolar fluid temperature, $\:\theta\:\left(\eta\:\right)$, and concentration, $\:\varphi\:\left(\eta\:\right)$, profiles at different values of the material parameter, $\:R$, given that $\:M=1$, $\:\text{P}\text{r}=0.7$, $\:\text{E}\text{c}=0.2$, $\:\text{S}\text{c}=\:0.22$, $\:s=0.5$, $\:{N}_{R}=3$, and $\:{k}_{r}=0.5$.

**Fig. 6:**
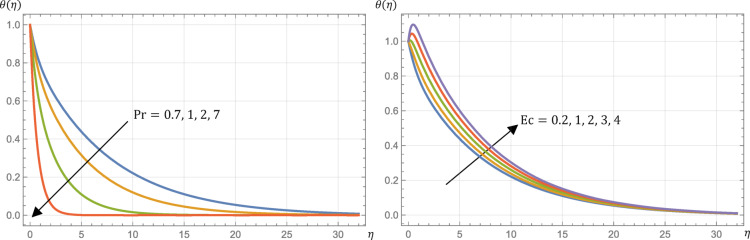
Micropolar fluid temperature, $\:\theta\:\left(\eta\:\right)$, profile at different Prandtl numbers, $\:\text{P}\text{r}$, where $\:\text{E}\text{c}=0.2$ beside $\:\theta\:\left(\eta\:\right)$ profile at different Eckert numbers, $\:\text{E}\text{c}$, where $\:\text{P}\text{r}=0.7$ if $\:M=1$, $\:R=s=0.5$, $\:\text{S}\text{c}=\:0.22$, $\:{N}_{R}=3$, and $\:{k}_{r}=0.5$.

**Fig. 7:**
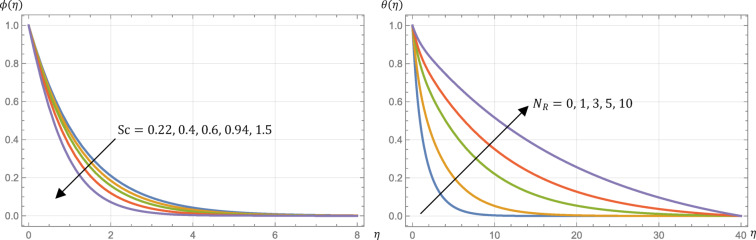
Micropolar fluid concentration, $\:\varphi\:\left(\eta\:\right)$, profile at different Schmidt numbers, $\:\text{S}\text{c}$, where $\:{N}_{R}=3$ beside temperature, $\:\theta\:\left(\eta\:\right)$, profile at different values of the radiation parameter, $\:{N}_{R}$, where $\:\text{S}\text{c}=0.22$ if $\:M=1$, $\:R=s=0.5$, $\:\text{P}\text{r}=\:0.7$, $\:\text{E}\text{c}=0.2$, and $\:{k}_{r}=0.5$.

**Fig. 8:**
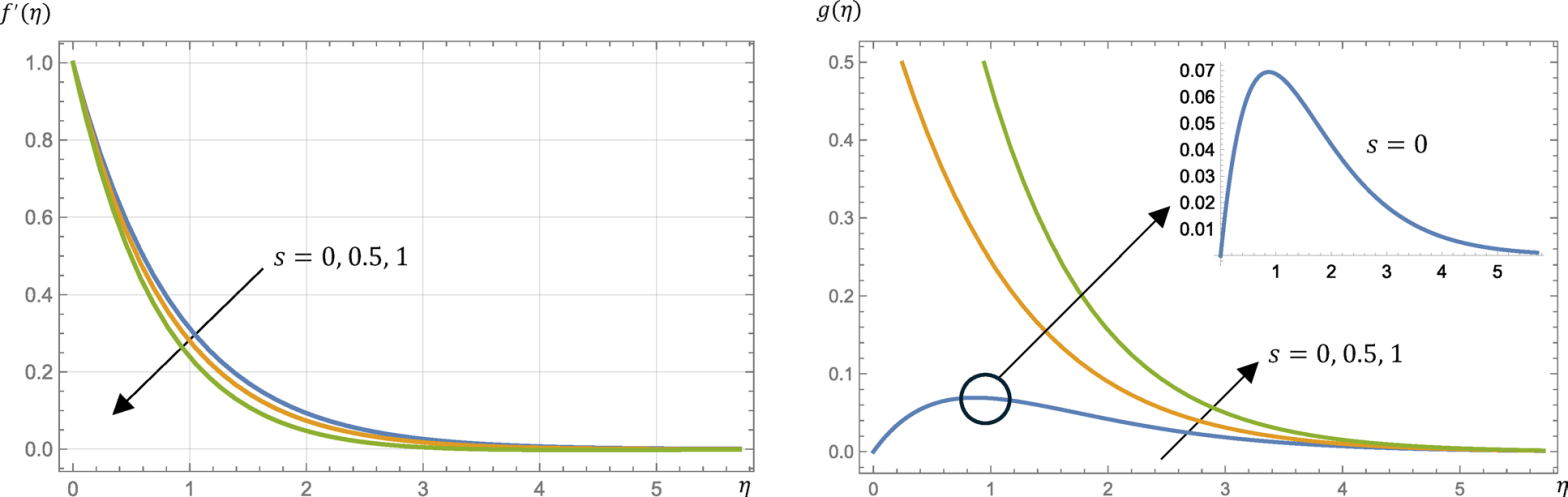
Micropolar fluid velocity, $\:{f}^{{\prime\:}}\left(\eta\:\right)$, and microrotation, $\:g\left(\eta\:\right)$, profiles at different values of the surface parameter, $\:s$, given that $\:M=1$, $\:\text{P}\text{r}=0.7$, $\:\text{E}\text{c}=0.2$, $\:\text{S}\text{c}=\:0.22$, $\:R=0.5$, $\:{N}_{R}=3$, and $\:{k}_{r}=0.5$.

**Fig. 9:**
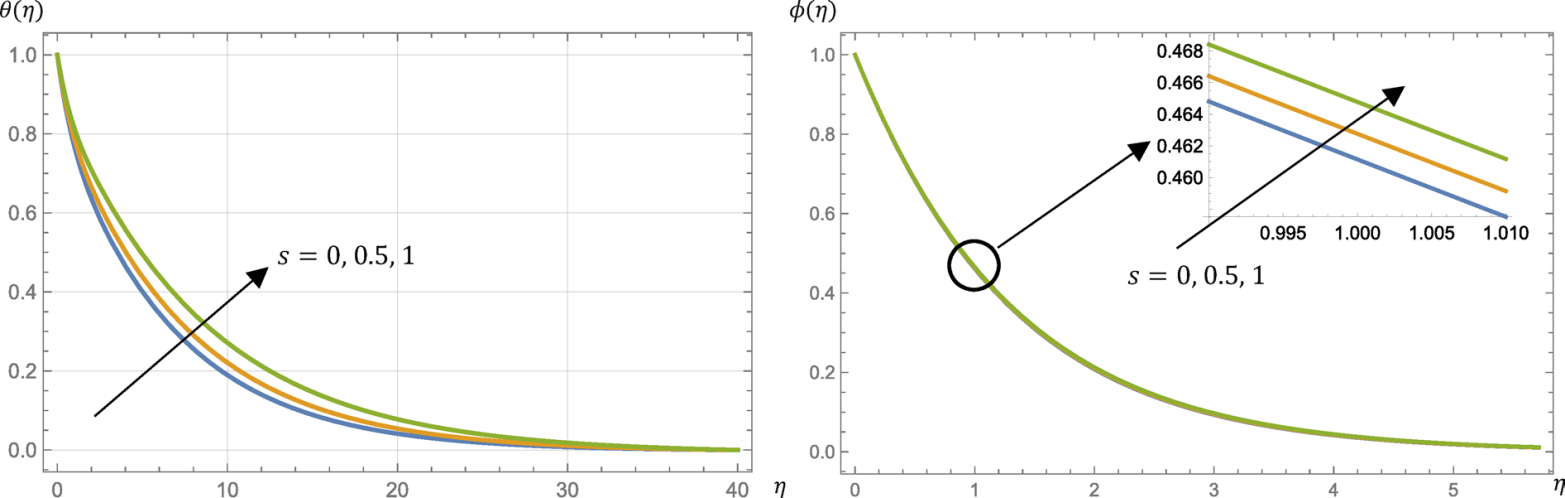
Micropolar fluid temperature, $\:\theta\:\left(\eta\:\right)$, and concentration, $\:\varphi\:\left(\eta\:\right)$, profiles at different values of the surface parameter, $\:s$, given that $\:M=1$, $\:\text{P}\text{r}=0.7$, $\:\text{E}\text{c}=0.2$, $\:\text{S}\text{c}=\:0.22$, $\:R=0.5$, $\:{N}_{R}=3$, and $\:{k}_{r}=0.5$.

**Fig. 10:**
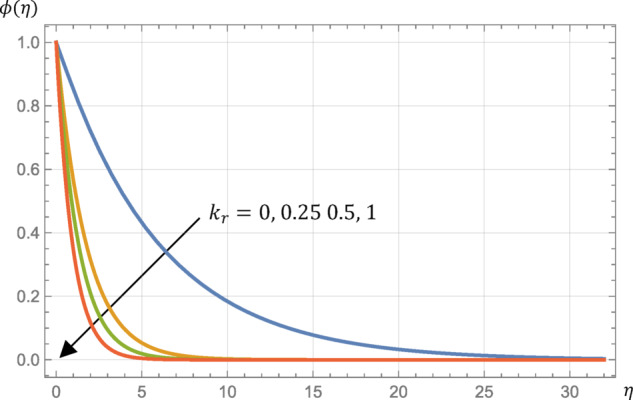
Micropolar fluid concentration, $\:\varphi\:\left(\eta\:\right)$, profile at different values of the chemical reaction parameter, $\:{k}_{r}$, given that $\:M=1$, $\:\text{P}\text{r}=0.7$, $\:\text{E}\text{c}=0.2$, $\:\text{S}\text{c}=\:0.22$, $\:R=0.5$, $\:{N}_{R}=3$, and $\:s=0.5$.

Table 1 presents a comparison with different methods presented by Aouadi^3^. The table shows a remarkably good harmony for diverse values of $\:M$. Table 2 introduces tabulated data for the modified skin friction, the modified Nusselt number, the modified Sherwood number, and the modified surface couple stress at different settings. Also, Figures (2–10) exhibit the micropolar fluid velocity, microrotation, temperature, and concentration profiles.

The magnetic parameter $\:M$ is dimensionless. It is defined as the ratio of the electromagnetic to the inertial forces. The introduction of a transverse magnetic field in an electrically conducting fluid gives rise to a retarding body force, which is known as the Lorentz force. An overview of the effects of the magnetic parameter $\:M$ can be found in Table 2. Physically, a magnetic field produces a Lorentz drag force that slows down the transition from laminar flow to turbulent flow, i.e., opposing the flow.

**Table 1:** Comparison with Aouadi^3^ at $\:R={N}_{R}={k}_{r}=0$, $\:\:\text{P}\text{r}=0.7$, $\:\text{E}\text{c}=0.02$, $\:\text{S}\text{c}=0.22$, and $\:\:s=1\:$for different values of $\:M$.

	$\:\varvec{M}$	FEM Aouadi [3]	ChFDM Aouadi [3]	Exact Aouadi [3]	Present result $\:\left(\varvec{n}=22\right)$
$\:-{\varvec{f}}^{\varvec{{\prime\:}}\varvec{{\prime\:}}}\left(0\right)$	0	1.000483	1.000483	1.000000	0.999993
	0.5	1.224742	1.224712	1.224776	1.224690
	1	1.414121	1.412166	1.412130	1.414020
	1.5	1.581141	1.581139	1.581138	1.580640
	2	1.732057	1.732051	1.732050	1.731020
$\:-{\varvec{g}}^{\varvec{{\prime\:}}}\left(0\right)$	0	1.000483	1.000483	1.000000	0.999993
	0.5	1.152392	1.152356	1.152361	1.152320
	1	1.261628	1.261668	1.261666	1.261380
	1.5	1.344946	1.344967	1.344930	1.344110
	2	1.410726	1.410746	1.410784	1.409160
$\:-{\varvec{\theta\:}}^{\varvec{{\prime\:}}}\left(0\right)$	0	1.064153	1.064234	1.064288	1.063910
	0.5	0.996628	0.996642	0.996632	0.995865
	1	0.940419	0.940473	0.940490	0.939382
	1.5	0.892573	0.892538	0.892505	0.891092
	2	0.850795	0.850738	0.850732	0.849048
$\:-{\varvec{\varphi\:}}^{\varvec{{\prime\:}}}\left(0\right)$	0	0.183578	0.183545	0.183412	0.183435
	0.5	0.157972	0.157949	0.157976	0.158092
	1	0.140844	0.140867	0.140851	0.141129
	1.5	0.128322	0.128382	0.128312	0.128805
	2	0.118620	0.118633	0.118619	0.119362

Hence, the skin friction is increased as shown in Table 2 and the velocity profile is decreased as exhibited in Fig. 2. Also from Fig. 2, it is found that as $\:M$ increases, the magnitude of the microrotation increases. Therefore, an enhanced transverse magnetic field controls the flow, which can be leveraged in material processing to influence the microstructural characteristics of the micropolar fluid. Ohmic heating, driven by electromagnetic work, causes an increase in the temperature profile of the fluid, which in turn results in a decrease in the surface heat flux, as demonstrated in Fig. 3 and presented in Table 2. Also, from Fig. 3, it is indicated that the concentration rises slightly with a growth in $\:M$. Finally, from Table 2, Sherwood number is decreased slightly but the surface couple’s stress is enhanced by growing the value of $\:M$.

The vortex viscosity, denoted as $\:k$, establishes a relationship between the average shear stress within the flow and the vertical velocity gradient. This coefficient quantifies the extent to which the behavior of micropolar fluids diverges from the Navier-Stokes equations. In scenarios where microrotation viscosity is absent, the linear momentum conservation law operates independently of microstructural influences. The micro-rotation viscosity $\:k$ serves as the dimensionless representation of the material parameter $\:R$.

Furthermore, this viscosity governs the angular rotation of the microstructure. Figure 4 presents the influences of the material parameter on the velocity and microrotation profiles. The velocity profile increases with increasing the value of $\:R$ while the microrotation profile decreases. Physically, the presence of larger $\:R$ values resulted in a reduction of the drag force in relation to a viscous fluid, thereby demonstrating that the micropolar fluid enhances the velocity of the fluid. Hence, by increasing the value of $\:R$, the skin friction is increased as demonstrated by Table 2. There is a decrease in the fluid temperature profile, so the surface heat flux (Nusselt number) increases as shown in Fig. 5; Table 2, respectively. Also, from Fig. 5, the concentration decreases slightly with an increase in $\:R$. Finally, from Table 2, Sherwood number and the surface couple’s stress are increased slightly by increasing the value of $\:R$.

The Prandtl number, abbreviated as $\:\text{P}\text{r}$, is characterized as a measure of the ratio of viscous diffusivity to thermal diffusivity. This property of fluids indicates the relative efficiency of momentum and thermal energy transport through diffusion in the respective velocity and thermal boundary layers. From Table 2, the surface shear stress (skin friction), Sherwood number, and the surface couple stress are not altered by any alteration in $\:\text{P}\text{r}$, so the fluid velocity, microrotation, and concentration are not disturbed too. The surface heat flux (Nusselt number) is increased by increasing $\:\text{P}\text{r}$. This phenomenon is elucidated by the fact that fluids characterized by a higher Prandtl number demonstrate diminished thermal conductivity, or, on the other hand, greater viscosity. Thereby, the fluid thermal profile will be slender and the surface heat flux will be highest as observed from Fig. 6; Table 2 for the higher value of $\:\text{P}\text{r}$.

The Eckert number, $\:\text{E}\text{c}$, is a dimensionless parameter that is instrumental in evaluating the relative significance of kinetic energy in heat transfer contexts. It is defined as the ratio of kinetic energy to the fluid’s enthalpy. The Eckert number serves to indicate the extent to which kinetic energy prevails overheat transfer, with higher values of $\:\text{E}\text{c}$ signifying a stronger influence of kinetic energy.

Furthermore, the product of the Eckert number and the Prandtl number, $\:\text{P}\text{r}$, is an essential factor in assessing the viscous dissipation of energy in flows characterized by low speeds. When the product $\:\text{E}\text{c}\:\text{P}\text{r}<<\:1$, the dissipation of energy can be disregarded in comparison to heat conduction in the fluid. In contrast, for large values of $\:\text{E}\text{c}\:\text{P}\text{r}$, energy dissipation becomes a critical consideration in the heat transfer process, with kinetic energy significantly affecting the temperature distribution and overall heat transfer. So, the fluid temperature profile is increased if $\:\text{E}\text{c}$ is increased as shown in Fig. 6 which in turn decreases the Nusselt number (surface heat flux) as reported in Table 2.

Physically, the increase in the Eckert number $\:\left(\text{E}\text{c}\right)$ leads to greater fluid friction, causing fluid particles to collide more frequently. This interaction produces heat energy, which ultimately reduces the Nusselt number as $\:\text{E}\text{c}$ increases. Table 2 confirms that the surface shear stress (skin friction), Sherwood number, and surface couple stress are unaffected by variations in $\:\text{E}\text{c}$, resulting in no changes to fluid velocity, microrotation, or concentration.

Schmidt number, $\:\text{S}\text{c}$, symbolizes the ratio between momentum diffusivity and the mass diffusivity. In other words, it represents how easily a fluid can transport momentum compared to the transport of mass. So, the fluid concentration is decreased by increasing Schmidt number as exhibited in Fig. 7.

It is essential to understand that the Sherwood number reflects the ratio of mass transfer due to convection relative to mass transfer due to diffusion. So, as reported by Table 2, Sherwood number is increased by enlarging Schmidt number. Also, from Table 2, the surface shear stress (skin friction), Nusselt number, and the surface couple’s stress are not altered by any alteration in $\:\text{S}\text{c}$, so the fluid velocity, temperature, and microrotation are not affected too.

The thermal radiation parameter, $\:{N}_{R}$, is instrumental in effectively controlling the thermal boundary layers. The effects of $\:{N}_{R}$ on surface shear stress (skin friction), surface heat flux (Nusselt number), and fluid temperature profiles are detailed in Table 2 and illustrated in Fig. 7. Increasing $\:{N}_{R}$ results in rising the surface heat flux (Nusselt number) and rising the fluid temperature profiles.

**Table 2:** The modified skin-friction, the modified Nusselt number, the modified Sherwood number, and the modified couple stress at different settings.

$\:M$	$\:R$	$\:\text{P}\text{r}$	$\:\text{E}\text{c}$	$\:\text{S}\text{c}$	$\:{N}_{R}$	$\:{k}_{r}$	$\:s$	$\:\frac{1}{2}{C}_{f}\sqrt{R{e}_{x}}$	$\:\frac{{\text{N}\text{u}}_{x}}{\sqrt{R{e}_{x}}}$	$\:-{\varphi\:}^{{\prime\:}}\left(0\right)$	$\:-\left(1+\frac{R}{2}\right){g}^{{\prime\:}}\left(0\right)$
0	0.5	0.7	0.2	0.22	3	0.5	0.5	1.1180	1.6269	0.7562	0.5000
0.5								1.3603	1.3791	0.7522	0.6155
1								1.5619	1.1963	0.7493	0.7120
1.5								1.7383	1.0536	0.7471	0.7963
2								1.8968	0.9377	0.7453	0.8720
1	0	0.7	0.2	0.22	3	0.5	0.5	1.4140	1.1417	0.7471	0.6307
	0.5							1.5619	1.1963	0.7493	0.7120
	1							1.7064	1.2518	0.7511	0.7450
	1.5							1.8421	1.3006	0.7526	0.7592
	3							2.2038	1.4113	0.7562	0.7921
1	0.5	0.7	0.2	0.22	3	0.5	0.5	1.5619	1.1963	0.7493	0.7120
		1						1.5619	1.6007	0.7493	0.7120
		2						1.5619	2.6917	0.7493	0.7120
		7						1.5619	5.8873	0.7493	0.7120
		10						1.5619	7.1579	0.7493	0.7120
1	0.5	0.7	0.2	0.22	3	0.5	0.5	1.5619	1.1963	0.7493	0.7120
			1					1.5619	0.5104	0.7493	0.7120
			2					1.5619	$\:-$0.3470	0.7493	0.7120
			3					1.5619	$\:-$1.2044	0.7493	0.7120
			4					1.5619	$\:-$2.0618	0.7493	0.7120
1	0.5	0.7	0.2	0.22	3	0.5	0.5	1.5619	1.1963	0.7493	0.7120
				0.4				1.5619	1.1963	0.7852	0.7120
				0.6				1.5619	1.1963	0.8262	0.7120
				0.94				1.5619	1.1963	0.8975	0.7120
				1.5				1.5619	1.1963	1.0155	0.7120
1	0.5	0.7	0.2	0.22	0	0.5	0.5	1.5619	0.8456	0.7493	0.7120
					1			1.5619	1.0386	0.7493	0.7120
					3			1.5619	1.1963	0.7493	0.7120
					5			1.5619	1.2740	0.7493	0.7120
					10			1.5619	1.4090	0.7493	0.7120
1	0.5	0.7	0.2	0.22	3	0	0.5	1.5619	1.1963	0.1522	0.7120
						0.25		1.5619	1.1963	0.5506	0.7120
						0.5		1.5619	1.1963	0.7493	0.7120
						1		1.5619	1.1963	1.0345	0.7120
1	0.5	0.7	0.2	0.22	3	0.5	0	1.4259	1.2917	0.7511	0.2641
							0.5	1.5619	1.1963	0.7493	0.7120
							1	1.3803	1.0622	0.7470	1.8671

The value of $\:{N}_{R}$ has no impact on the surface skin friction, the surface couple stress, and Sherwood number, thereby, the velocity, the microrotation, and the concentration boundary layers have not been influenced by any change in $\:{N}_{R}$.

The physical findings agree with expectations, as the increase in $\:{N}_{R}$ signifies a decrease in the Rosseland radiation absorptivity, $\:{\alpha\:}^{*}$. It is known that the divergence of the radiative heat flux, $\:\partial\:{q}_{r}/\partial\:y$, intensifies as $\:{\alpha\:}^{*}$ decreases, thereby increasing the rate of radiative heat transfer to the fluid and consequently raising the fluid temperature. Also, it is marked that the domain of temperature changes due to Prandtl number in the existence of thermal radiation is higher than without it.

The surface parameter, $\:s$, takes different values; $\:s=0$ (no slipping mode) and $\:0<s\le\:1$ (slipping mode). From Figs. 8 and 9, the fluid velocity is decreased while the fluid microrotation, temperature, and concentration are increased by changing the modes from no slipping mode to slipping one.

From Table 2, the skin friction coefficient is increased if $\:s$ is increased from $\:0$ to $\:0.5$ while the reverse happened if $\:s$ is increased from $\:0.5$ to $\:1$. The surface couple stress is increased by increasing the value of $\:s$ but Nusselt and Sherwood numbers are decreased.

The surge in the chemical reaction suggests that the rate of the reaction at the fluid-surface interface is accelerating. When the reaction rate is significantly high, due to an increased chemical reaction parameter $\:\left({k}_{r}\right)$, the reactants are consumed at a faster rate at the surface, resulting in a steeper concentration gradient close to the surface. A higher chemical reaction parameter facilitates a more rapid reaction at the surface. This rapid reaction at the surface depletes the concentration of reactants in the vicinity more quickly and leads to a reduction in the thickness of the concentration profile, with the concentration of reactants declining more swiftly within this layer, as shown in Fig. 10. Hence, Sherwood number is increased by enhancing $\:{k}_{r}$ as seen in Table 2. On the other hand, Table 2 reports that the surface skin friction, Nusselt number, and the surface couple’s stress are not altered by any alteration in $\:{k}_{r}$ so the velocity, the microrotation, and the temperature profiles have not been influenced by any change in $\:{k}_{r}$.

## Conclusion

In this study, the Legendre-collocation method is used to investigate the effect of magnetic field, thermal radiation, and chemical reaction on the boundary layer flow over a steady stretching surface in a micropolar fluid associated with heat and mass transfer. A comparison with previously published work was performed and the results were found to be in an excellent agreement. The effects of different parameters on the flow, temperature, micro-rotation, and concentration profiles and on the local skin friction, Nusselt number, local surface couple stress, and Sherwood number are carried out. It is found that:

1. The results of the present study are in good agreement with the results available in the literature^2^, which suggests the validity of the present model.
2. Elevating the material parameter leads to an increase in velocity, while simultaneously causing a reduction in the maximum angular velocity, fluid temperature, and fluid mass concentration. See Figs. 4 and 5.
3. Increasing Prandtl number decreases the fluid temperature, and this effect is increased when the fluid is subjected to a thermal radiation. Hence, Increasing the intensity of the radiation increases the temperature. Also, increasing Eckert number increases the temperature. See Figs. 6 and 7.
4. Increasing the chemical reaction parameter decreases the fluid mass concentration. Also, increasing Schmidt number decreases concentration. See Figs. 7 and 10.
5. The skin friction and the surface couple stress are decreased with decreasing the magnetic field or the material parameter as listed in Table 2.
6. The local Nusselt number is increased with increasing the material parameter, Prandtl number, or the thermal radiation or with decreasing the magnetic parameter or Eckert number as listed in Table 2.
7. The local Sherwood number decreases with increasing the magnetic parameter or with decreasing the material parameter, Schmidt number, or the chemical reaction as listed in Table 2.

## Data Availability

No publicly available repositories or databases are suitable for the current data submission. All data supporting the results of this study are available in the article. They can also be obtained from the corresponding author, MF, upon reasonable request.

## List of symbols

*B*: magnetic field strength $(kg/s^2 A)$
c_p_: specific heat$$(J/kg.K)$$
*C*: mass concentration$$(kg/m^3 )$$
*C*_*f*_: local skin friction coefficient
*C*_R_: surface concentration excess ratio
*D*: coefficient of mass diffusivity
Ec: Eckert number
*f*: dimensionless stress
*g*: dimensionless microrotation
*k*_*r*_: chemical reaction rate
*K*: vortex viscosity $$(kg/m.s)$$
*m*_*x*_: surface couple stress
*M*: magnetic parameter
*M*_*x*_: dimensionless surface couple stress
*N*: microrotation/angular velocity (s^-1^)
*N*_*R*_: thermal radiation parameter
Nu_*x*_: local Nusselt number
Pr: Prandtl number
q_*m*_: surface mass flux$(kg/m^2 s)$
q_*r*_: radiative heat flux$(W/m^2 )$
q_*w*_: surface heat flux$(W/m^2 )$
*R*: material parameter
Re_*x*_: local Reynolds number
*s*: surface parameter
Sc: Schmidt number
Sh_*x*_: local Sherwood number
*T*: temperature (K)
*T*_*R*_: surface temperature excess ratio
*U*: stretching velocity (m/s)
(*u*,*w*): velocity components (m/s)
(*x*,*y*): cartesian coordinates (m)

## *Greek letters*

$$\alpha$$: thermal diffusivity (m^2^/s)
$\alpha^{*}$: Rosseland radiation absorptivity
$\gamma$: spin gradient viscosity (kg/m. s)
$$\eta$$: dimensionless variable
$$\theta$$: dimensionless temperature
$$\kappa$$: thermal conductivity (W/m. K)
$\mu$: dynamic viscosity (kg/m. s)
$$\nu$$: kinematic viscosity (m^2^/s)
$\xi$: microinertia density (kg/m^3^)
$\rho$: fluid density (kg/m^3^)
$$\sigma$$: electrical conductivity $$(A^2 s^3/m^3 kg)$$
$\sigma^*$: Stefan-Boltzmann constant
$\tau_{w}$: surface shear stress (kg/m. s)
$$\phi$$: dimensionless concentration

## *Superscript*

$\prime$: Differentiation with respect to $\eta$

## *Subscripts*

*w*: stretching surface conditions
0: reference fluid conditions
$$\infty$$: ambient conditions
